# Quantitative proteomic analysis of the *Salmonella*-lettuce interaction

**DOI:** 10.1111/1751-7915.12114

**Published:** 2014-02-11

**Authors:** Yuping Zhang, Renu Nandakumar, Shannon L Bartelt-Hunt, Daniel D Snow, Laurie Hodges, Xu Li

**Affiliations:** 1 Department of Civil Engineering, University of Nebraska-LincolnLincoln, NE, 68588, USA; 2 Proteomics and Metabolomics Core Facility, Redox Biology Center, Department of Biochemistry, University of Nebraska-LincolnLincoln, NE, 68588, USA; 3 School of Natural Resources, University of Nebraska-LincolnLincoln, NE, 68588, USA; 4 Deptartment of Agronomy & Horticulture, University of Nebraska-LincolnLincoln, NE, 68588, USA

## Abstract

Human pathogens can internalize food crops through root and surface uptake and persist inside crop plants. The goal of the study was to elucidate the global modulation of bacteria and plant protein expression after *Salmonella* internalizes lettuce. A quantitative proteomic approach was used to analyse the protein expression of *Salmonella enterica* serovar Infantis and lettuce cultivar Green Salad Bowl 24 h after infiltrating *S*. Infantis into lettuce leaves. Among the 50 differentially expressed proteins identified by comparing internalized *S*. Infantis against *S*. Infantis grown in Luria Broth, proteins involved in glycolysis were down-regulated, while one protein involved in ascorbate uptake was up-regulated. Stress response proteins, especially antioxidant proteins, were up-regulated. The modulation in protein expression suggested that internalized *S*. Infantis might utilize ascorbate as a carbon source and require multiple stress response proteins to cope with stresses encountered in plants. On the other hand, among the 20 differentially expressed lettuce proteins, proteins involved in defense response to bacteria were up-regulated. Moreover, the secreted effector PipB2 of *S*. Infantis and R proteins of lettuce were induced after bacterial internalization into lettuce leaves, indicating human pathogen *S*. Infantis triggered the defense mechanisms of lettuce, which normally responds to plant pathogens.

## Introduction

Outbreaks of diseases associated with contamination of fresh produce by human pathogens have increased in the past decades (Lynch *et al*., 2009; Schikora *et al*., 2012). Better practice during postharvest processing or the use of a terminal control such as disinfection could reduce the load of microorganisms on the surfaces of fresh produce. However, concerns are raised over food crops contaminated with human pathogens that get internalized in plants during field production, because washing or disinfection may not be effective to remove the internalized bacteria (Wei *et al*., 1995; Weissinger *et al*., 2000).

Human pathogens can internalize into plants through root or leaf uptake. *Salmonella enterica* serovars Cubana and Dublin can accumulate inside hydroponically grown alfalfa and lettuce, respectively, through root uptake both at the level of 4 log CFU/g fresh weight (Dong *et al*., 2003; Klerks *et al*., 2007,2007). Internalization of human pathogens can also occur through root uptake when the pathogens are introduced by contaminated soil or irrigation water (Wachtel *et al*., 2002; Hora *et al*., 2005; Klerks *et al*., 2007,2007). A recent study on leaf uptake shows that spray irrigation with contaminated water can lead to the internalization of *Escherichia coli* O157:H7 into spinach leaves (Erickson *et al*., 2010).

The fate of human pathogens inside plants is determined by their interaction with plants. Schikora and colleagues (2001) found that *Salmonella enterica* serovar Typhimurium infiltrated into *Arabidopsis* leaves multiplied within the first 2 days after infiltration and remained viable for at least 4 days. *Escherichia coli* O157:H7 could survive inside spinach leaves for up to 14 days after spray inoculation (Erickson *et al*., 2010). In contrast, internalized *S*. Newport could not be detected in basil leaves 22 h after introducing the bacteria by placing cut petiole in a bacteria suspension (Gorbatsevich *et al*., 2013). Despite the important findings reported in these studies, it is still poorly understood how internalized human pathogens adjust their metabolism to survive inside plants.

In recent years, mRNA-based transcriptomic approaches have been used to examine the gene expression of human pathogens living in and on plants. After spray-inoculated on lettuce leaf surface for 1–3 days, *E. coli* K-12 and O157:H7 up-regulated genes associated with starvation and curli production (Fink *et al*., 2012). Similarly, *E. coli* K-12 cells that were attached to and internalized inside the lettuce root up-regulated genes involved in attachment, stress responses and protein synthesis (Hou *et al*., 2012). After 15–30 min of exposure to lettuce leaf lysates, *E. coli* O157:H7 up-regulated its flagellar machinery, fimbrial, type III secretion system (T3SS) (a virulent factor) and stress response (especially oxidative stress) genes (Kyle *et al*., 2010). Collectively, these observations suggest that human pathogens encounter stresses in plants and the temporal changes in the expression of certain genes depend on the location of the bacteria (i.e. outside or inside the plant root or leaf). Despite the important information gained from these transcriptomic studies, it should be noted that the expressional levels of mRNA and proteins are not directly proportional and transcriptomics cannot detect post-translational modifications on proteins (Abbott, 1999).

Different from transcriptomics, proteomics directly studies the ultimate products of gene expression. Although the advantages of using proteomics to study bacterial adaptation to plant-associated environments has been recognized (Knief *et al*., 2011), the application of proteomics in studying the interaction between human pathogens and plants has been limited. The objective of this study was to investigate the proteomic responses of *Salmonella* after internalizing lettuce leaf and the proteomic responses of lettuce leaf to internalized *Salmonella*. Two-dimensional nanoliquid chromatography-tandem mass spectrometry (2D nano LC-MS/MS) approach was utilized for the quantitative shotgun proteomic analysis. Two comparisons were made: global proteome profiles of internalized *Salmonella versus Salmonella* grown in Luria Broth (LB), and proteome profiles of lettuce leaf containing internalized *Salmonella versus* lettuce leaf without internalized *Salmonella*.

## Results and discussion

### Quantitative proteomic analysis

Leaves of 5-week old leafy lettuce (*Lactuca sativa*) cultivar Green Salad Bowl were inoculated by *Salmonella enterica* serovar Infantis (*S*. Infantis) suspension using syringe infiltration (Katagiri *et al*., 2002). Because previous experiences with syringe infiltration showed only a fraction of the cells in a bacterial suspension could end up in leaves, 300 μl of 10^10^ CFU/ml bacterial culture (stationary phase *S*. Infantis grown in Luria Broth), which was washed and re-suspended in sterile water, was infiltrated to ensure sufficient internalized *S*. Infantis cells to elicit significant proteomic response. Control plants were infiltrated with the same amount of sterile water. Two biological replicates were included in each group (i.e. treatment and control groups). Lettuce leaves were harvested 24 h after infiltration, and bacteria on the leaf surface were removed by sonication and vortexing for 4 times. Bacterial and plant proteins in lettuce leaf samples containing internalized *S*. Infantis were separated (details in Supporting Information). In addition, bacterial protein was extracted from stationary phase *S*. Infantis grown in LB and plant proteins from lettuce leaf without internalized *S*. Infantis. Protein digestion and 2D LC-MS/MS analysis were performed as previously described in the literature (Nandakumar *et al*., 2011; Li *et al*., 2012). The acquired MS/MS spectra from the bacterial protein samples were searched against the S. Typhimurium 14028S database (5323 sequences), and those from the lettuce protein samples against both the *Lactuca sativa* expressed sequence tag (EST) database (128172 sequences) and a custom-made database including *Lactuca sativa* protein sequences (1506 entries) on NCBI (Cho *et al*., 2009). The criteria for protein identification included the detection of at least one unique peptide per protein and a protein probability score of ≥90%. Relative quantitation of proteins was done by using the label-free method of spectral counting (Liu *et al*., 2004) with the normalized spectral counts for each protein. Proteins having ≥ 2-fold change in abundance (*P* ≤ 0.05) were considered as differentially expressed. More details about the methods can be found in Supporting Information.

### Protein expression profile of *S*. Infantis

The protein expression profile was compared between the *S*. Infantis internalized in lettuce leaves (i.e. 24 h after infiltration) and the stationary-phase *S*. Infantis grown in LB medium (i.e. immediately before infiltration). A total of 541 proteins were detected, and 50 proteins were differentially expressed (≥ 2-fold and *P* < 0.05), among which 34 proteins were up-regulated and 16 were down-regulated (Table 1).

**Table 1:** Proteins that were differentially expressed in *S*. Infantis after internalization into lettuce

Protein name	Uniprot Accession	Gene	Fold change	*P*-value	# of unique peptides
***Metabolism***					
*Carbon*					
Putative PTS system, ascorbate-specific IIB component	D0ZQJ7		37.0	<1.0E-04	2
Alcohol dehydrogenase	D0ZXP4	*adhP*	−5.9	<1.0E-04	7
Phosphopyruvate hydratase	D0ZVP5	*eno*	−8.8	<1.0E-04	7
*Amino acid*					
Tryptophan synthase subunit alpha	D0ZIZ5	*trpA*	20.6	<1.0E-04	1
Aspartate-semialdehyde dehydrogenase	D0ZJH4	*asd*	5.3	<1.0E-04	1
Putative aspartate racemase	D0ZU78		5.0	<1.0E-04	1
*Nucleotide*					
Allantoinase	D0ZP18	*allB*	3.5	3.4E-02	2
Cytidylate kinase	D0ZSI5	*cmk*	6.5	5.1E-04	1
Dihydroorotase	D0ZUK7	*pyrC*	20.5	<1.0E-04	1
*Lipid*					
Acyl carrier protein	D0ZUP3	*acpP*	2.0	<1.0E-04	3
*Cofactors and vitamins*					
Adenosylcobinamide kinase	D0ZMB8	*cobU*	4.0	1.7E-02	1
**Protein synthesis**					
50S Ribosomal protein L13	D0ZY47	*rplM*	4.5	8.8E-03	2
Transcriptional regulator	D0ZR74		10.5	<1.0E-04	1
tRNA-dihydrouridine synthase C	D0ZNJ3	*yohl*	6.0	1.1E-03	2
DNA binding protein	D0ZU24	*stpA*	4.0	1.7E-02	1
23S rRNA 5-methyluridine methyltransferase	D0ZVQ3	*rlmD*	4.0	1.7E-02	1
30S Ribosomal subunit S22	D0ZXP1	*rpsV*	-5.7	1.9E-02	3
**Pathogen-associated molecular patterns (PAMPs)**					
Flagellin	D0ZL85	*fliC*	-6.0	1.2E-02	4
Elongation factor Tu	D0ZIM1	*tuf_1*	-2.1	3.2E-03	9
Lipid A biosynthesis lauroyl acyltransferase	D0ZUJ8	*htrB*	5.8	<1.0E-04	1
**Stress response**					
Superoxide dismutase	D0ZWV7	*sodB*	7.0	2.4E-04	1
Superoxide dismutase	D0ZWW6	*sodC_2*	2.5	6.3E-03	2
Putative thiol-alkyl hydroperoxide reductase	D0ZMY2		2.0	2.7E-03	3
Bacterioferritin, iron storage and detoxification protein	D0ZIL8	*bfr*	7.0	2.4E-04	1
NAD(P)H dehydrogenase (quinone)	D0ZTQ2	*wraB*	3.3	1.2E-02	3
Putative intracellular proteinase	D0ZXW4	*yhbO*	−25.0	<1.0E-04	4
Chaperonin	D0ZS62	*groL*	−2.5	2.6E-02	
Thioredoxin	D0ZNP5	*trxA*	−8.7	1.3E-04	7
Transcriptional regulator HU subunit alpha	D0ZQX4	*hupA*	−2.5	<1.0E-04	5
Hypothetical protein STM14_1832	D0ZXI6	*ydeI*	−11.0	<1.0E-04	3
**Cell envelope**					
dTDP-Glucose 4,6-dehydratase	D0ZNQ1	*rffG*	5.5	2.2E-03	1
**Transport**					
Hypothetical protein STM14_1021	D0ZS98	*ybjL*	6.0	1.1E-03	3
Sodium/panthothenate symporter	D0ZIF6	*panF*	4.5	8.8E-03	2
Low affinity gluconate transporter	D0ZJH7	*gntU*	2.0	5.7E-03	1
Putative ABC-type multidrug transport system ATPase component	D0ZKD9	*yhiH*	3.5	3.4E-02	1
**Unknown**					
Hypothetical protein STM14_0531	D0ZN35		5.5	2.2E-03	1
Phage tail component H-like protein	D0ZST4		2.2	1.9E-02	2
Putative cytoplasmic protein	D0ZXI3		6.5	<1.0E-04	1
Putative cytoplasmic protein	D0ZIZ8	*yciE*	2.0	<1.0E-04	9
Hypothetical protein STM14_2884	D0ZQJ3	*yfcC*	6.0	1.1E-03	1
Hypothetical protein STM14_3293	D0ZTU4	*yfjG*	4.0	1.7E-02	1
Hypothetical protein STM14_4694	D0ZMW1	*yifE*	2.4	1.7E-02	1
Putative type II restriction enzyme methylase subunit	D0ZU54		5.5	2.2E-03	1
Hypothetical protein STM14_0428	D0ZM36	*yahO*	−5.7	1.9E-02	2
Hypothetical protein STM14_0454	D0ZM62	*psiF*	−3.9	7.5E-04	4
Putative cytoplasmic protein	D0ZVJ6		−14.0	1.0E-04	2
Hypothetical protein STM14_1588	D0ZW41	*spy*	−5.4	2.8E-02	3
Putative cytoplasmic protein	D0ZJ87		−6.3	7.9E-03	1
Hypothetical protein STM14_4278	D0ZJJ1	*yhhA*	−8.8	2.1E-04	2

#### Metabolism

The most significant change among all differentially expressed bacterial proteins, a 37-fold increase, was seen in a putative cytoplasmic protein. The protein is determined to be an ascorbate-specific IIB component and is believed to phosphorylate ascorbate during transmembrane transport. Interestingly, ascorbate (vitamin C) is abundant in lettuce leaf [9.2 mg/100 g (USDA, 2013)], and *Salmonella* has been reported to be capable of consuming ascorbate when its preferred carbon sources are not available (Eddy and Ingram, 1953).

**Fig 1:**
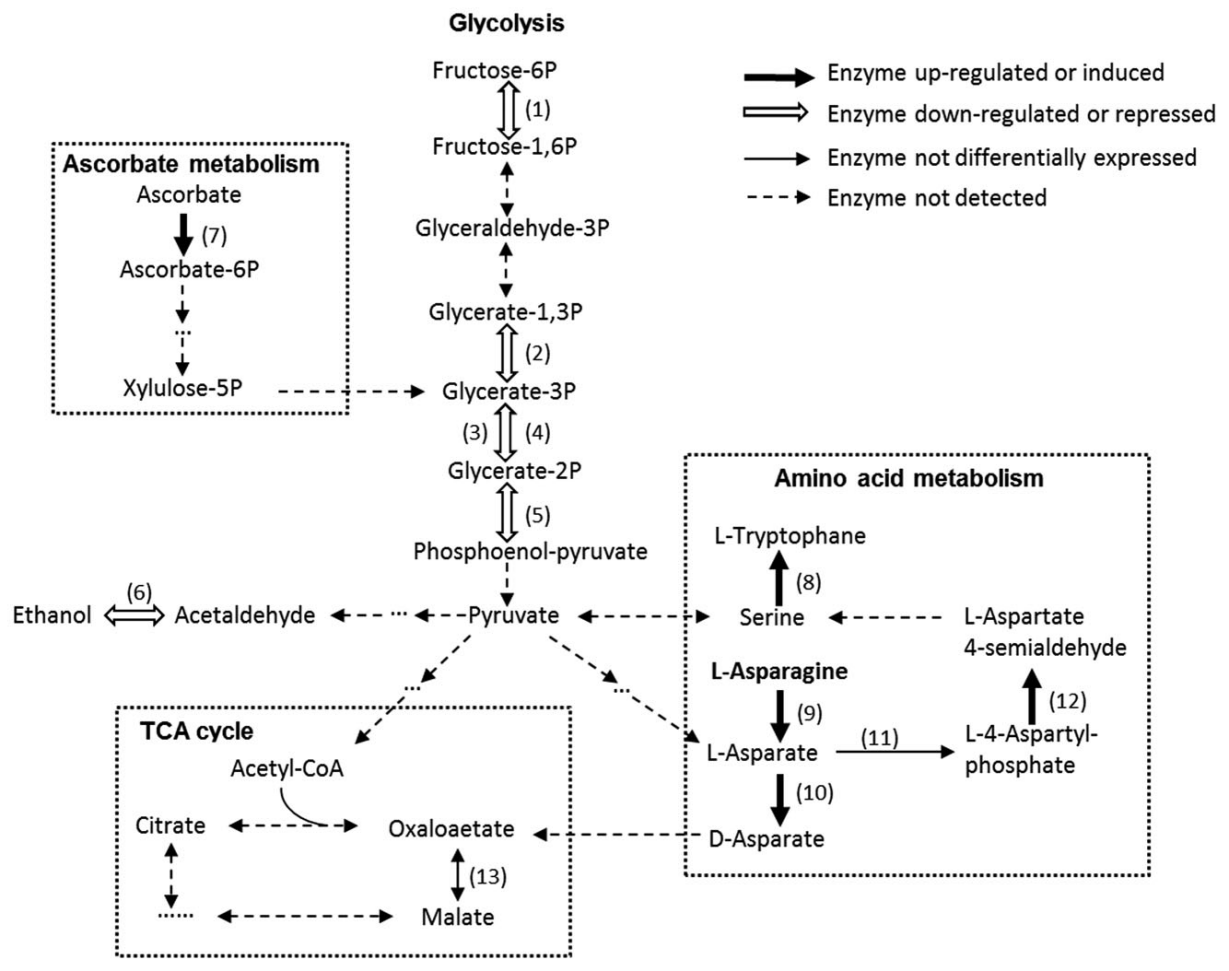
Changes in selective metabolic pathways (i.e. glycolysis, amino acid metabolism, ascorbate metabolism and TCA) of *Salmonella* internalized in lettuce leaves compared to *Salmonella* grown in LB. Proteins shown are: (1) 6-phosphofructokinase; (2) phosphoglycerate kinase; (3) 2,3-bisphosphoglycerate-independent phosphoglycerate mutase; (4) 2,3-bisphosphoglycerate-dependent phosphoglycerate mutase; (5) phosphopyruvate hydratase; (6) alcohol dehydrogenase; (7) putative PTS system, ascorbate-specific IIB component; (8) tryptophan synthase subunit alpha; (9) L-asparaginase; (10) putative aspartate racemase; (11) aspartate kinase; (12) aspartate-semialdehyde dehydrogenase; (13) malate dehydrogenase.

Phosphopyruvate hydratase and alcohol dehydrogenase, two enzymes involved in glycolysis, were down-regulated 8.8- and 5.9-fold respectively. In addition, several enzymes involved in glycolysis (i.e. 6-phosphofructokinase, phosphoglycerate kinase and phosphoglycerate mutases) were detected only in the *Salmonella* grown in LB but not in the *Salmonella* grown in lettuce leaves (Fig. 1). Glycolysis starts with glucose and fructose, which are present in leaf lettuce at the levels of 0.36 g and 0.43 g per 100 g respectively (USDA, 2013). The decrease in the abundance of multiple enzymes involved in glycolysis suggests that these monosaccharides may not be available to *Salmonella* inside lettuce leaves. Alternatively, internalized *Salmonella* may utilize less preferred but available substrates, such as ascorbate. In plants, the level of ascorbate increases under stress conditions, such as pathogen invasion (Noctor and Foyer, 1998).

#### Stress response

Stress response proteins accounted for a major class of the differentially expressed proteins (Table 1). Several proteins involved in response to oxidative stress were up-regulated. Superoxide dismutase (SodC_2, up-regulated 2.5-fold) is a periplasmic or membrane-associated protein in several gram-negative bacteria, and protects bacteria from extracellular reactive oxygen species (ROS) (Battistoni, 2003). Another superoxide dismutase (SodB, up-regulated 7-fold) is an intracellular protein, and removes ROS produced by aerobic metabolism (Farrant *et al*., 1997). Bacterioferritin, an iron storage and detoxification protein (Bfr, up-regulated 7-fold) is the major Fe storage protein in *S*. Typhimurium. It sequesters Fe to prevent generating highly toxic hydroxyl radical (Fe^2+^ + H_2_O_2_ → Fe^3+^ + OH^−^ + OH^•^) when Fe is in excess and releases Fe when exogenous Fe is limiting (Velayudhan *et al*., 2007). *Salmonella bfr* mutants appeared to be more susceptible to oxidative stress than the wild type (Velayudhan *et al*., 2007). Under the control of the central regulator of general stress responses RpoS (Patridge and Ferry, 2006), NAD(P)H dehydrogenase (quinone) (WraB, up-regulated 3.3-fold) is often up-regulated under stresses such as acid, salt and H_2_O_2_ (Pomposiello *et al*., 2001; Tucker *et al*., 2002; Cheung *et al*., 2003). Finally, putative thiol-alkyl hydroperoxide reductase (up-regulated 2.0-fold) is an antioxidant, which can scavenge H_2_O_2_ and enhance oxidative stress resistance (Hebrard *et al*., 2009). Because generating ROS is a universal defensive strategy employed by plants when challenged by pathogenic or beneficial bacteria (Shetty *et al*., 2008), it is not surprising that internalized *Salmonella* up-regulated multiple proteins to resist ROS.

Interestingly, about half of the stress response proteins that were differentially expressed were down-regulated in internalized *Salmonella* (Table 1). Chaperonin (GroL, down-regulated 2.5-fold) refolds and assembles unfolded polypeptides (Sherman and Goldberg, 1992), and is essential in cell growth and survival under heat and acid stresses (Baumann *et al*., 1996; Hartke *et al*., 1997). Genes coding for transcriptional regulator (HupA, down-regulated 2.5-fold) and putative intracellular proteinase (YhbO, down-regulated 25-fold) can increase the survival of *S*. Typhimurium under the exposure to artificial sea water (Haznedaroglu, 2010), and the latter can act in response to oxidative, thermal, UV and pH stresses (Abdallah *et al*., 2007).

#### Pathogen associated molecular patterns (PAMPs)

PAMPs from bacteria can be recognized by host plants and can trigger plants' basal defense responses. Known PAMPs include flagellin, lipopolysaccharide (LPS) and elongation factor Tu (EF-Tu) (Chisholm *et al*., 2006; Zipfel, 2008). In this study, flagellin and EF-Tu were down-regulated, while lipid A biosynthesis lauroyl acyltransferase (*htrB*) involved in LPS biosynthesis was up-regulated (Table 1).

Although flagella, which are composed of flagellin, facilitate *Salmonella* to move toward plant roots or attach to plant leaf surface (Cooley *et al*., 2003; Kroupitski *et al*., 2009), they provide little benefit to endophytic bacteria because the endophytes are usually nonmotile upon entering plants (Hattermann and Ries, 1989; Kamoun and Kado, 1990). Studies reported that flagella mutants of *E. coli* O157:H7 and *Salmonella* could survive better in *Arabidopsis* and in alfalfa (*Medicago sativa*), respectively, than respective wild types (Iniguez *et al*., 2005; Seo and Matthews, 2012), suggesting that the down-regulation of flagellin may increase the fitness of human pathogens in plants.

#### Type III secretion system (T3SS)

In addition to the differentially expressed proteins reported in Table 1, secreted effector protein (PipB2) was detected in internalized *Salmonella* but not in LB-grown *Salmonella*. PipB2 can be secreted via T3SS-2, which is often expressed after *Salmonella* has entered an epithelial cell or a macrophage. T3SS-1 enables bacterial invasion of epithelial cells, and T3SS-2 enhances bacterial survival and replication in epithelial cells (Waterman and Holden, 2003). A recent study demonstrated that *Salmonella* could suppress the immune system of *Arabidopsis* plants using T3SS-1 and T3SS-2 (Schikora *et al*., 2011).

### Protein expression profile of lettuce

Two databases were used to identify lettuce proteins: expressed sequence tag (EST) sequences of *Lactuca sativa* from CGPDB and a custom-built database comprising *Lettuce* protein sequences available in NCBI (Cho *et al*., 2009). A total of 289 lettuce proteins were identified using the EST database with 174 and 189 proteins detected in lettuce without and with internalized *Salmonella*, respectively. Because lettuce sequences in the EST database are not annotated, the sequence hits from the EST database were blasted against the proteins of *Arabidopsis thaliana* for functional information (Cho *et al*., 2009). Among the lettuce proteins that are homologous to *A. thaliana* proteins, 17 proteins were up-regulated and 3 were down-regulated (Table 2). Using the custom-built lettuce protein database, among the 163 proteins identified, 25 proteins were detected only in lettuce with internalized *Salmonella* but not in control lettuce (Supporting Information, Table S1).

**Table 2:** Proteins that were differentially expressed in lettuce after internalization of *S*. infantis

Protein name	Uniprot Accession	Gene	Fold change	*P*-value	# of unique peptides
Pyruvate dehydrogenase E1 subunit beta-1	Q38799	PDH2	7.5	<1.0E-04	2
Triosephosphate isomerase	P48491	CTIMC	2.3	1.9E-02	2
Fructose-bisphosphate aldolase 1	F4IGL7	FBA1	−7.2	5.8E-03	1
2-cys Peroxiredoxin	Q96291	BAS1	10.0	5.5E-04	1
Actin 4	P53497	ACT12	3.5	2.4E-02	1
Nucleoside diphosphate kinase	P39207	NDPK1	2.9	2.8E-02	1
Ribulose bisphosphate carboxylase/oxygenase activase	F4IVZ7	RCA	−4.8	4.1E-02	2
Selenoprotein, Rdx type	Q8W1E5	AT5G58640	4.0	3.6E-03	1
Superoxide dismutase [Cu-Zn] 1	P24704	CSD1	4.3	1.2E-02	2
calmodulin 5	P59220	CAM7	5.7	2.4E-02	1
Plasma membrane-associated cation-binding protein 1	Q96262	PCAP1	3.3	3.0E-02	1
Oxygen-evolving enhancer protein 3-2	Q41932	PSBQ2	2.9	<1.0E-04	3
Oxygen-evolving enhancer protein 1–2	Q9S841	PSBO2	3.5	2.4E-02	2
Two-component response regulator-like APRR2	Q6LA43	APRR2	2.3	4.5E-03	1
30S Ribosomal protein S31, chloroplastic	O80439	RPS31	3.9	2.3E-03	1
Ferredoxin-NADP reductase, leaf-type isozyme 2	Q8W493	LFNR2	2.7	6.8E-04	2
40S Ribosomal protein S8-2	Q9FIF3	RPS8B	6.0	5.5E-04	1
photosystem I reaction center subunit 2-2	Q9S714	PSAE2	4.0	1.2E-02	2
50S Ribosomal protein L12-1, chloroplastic	P36210	RPL12A	4.1	4.8E-02	2
Purple acid phosphatase 13	Q9SIV9	PAP10	−4.8	<1.0E-04	1

Several lettuce proteins were up-regulated in response to *S*. Infantis internalization (Table 2). Pyruvate dehydrogenase E1 subunit beta-1 (up-regulated 7.5-fold) is considered a PAMP-responsive protein. It increased in abundance when *Arabidopsis* was challenged by a *hrpA* mutant of *Pseudomonas syringae*, which could only activate plant basal defense (Jones *et al*., 2006; Jones and Dangl, 2006). 2-cys Peroxiredoxin (up-regulated 10-fold) may play a role in defense-related redox signaling, because it can reduce reactive nitrogen peroxides generated during incompatible interactions during which the host resists to bacteria and no disease develops (Jones *et al*., 2004). Superoxide dismutase [Cu-Zn] 1 was up-regulated 4.3 fold, possibly as a self-protective antioxidant response to the plant ROS induced by internalized *Salmonella* (Jagadeeswaran *et al*., 2009)*.* Ferredoxin–NADP reductase, which was up-regulated 2.7-fold following *Salmonella* internalization, plays a key role in regulating the relative amounts of cyclic and non-cyclic electron flow to meet plant demand for ATP and reducing power (Hanke *et al*., 2005; Lintala *et al*., 2007). Its involvement in defense response to bacteria has been inferred from computational annotation and expression patterns (Jones *et al*., 2006; Jones and Dangl, 2006; Heyndrickx and Vandepoele, 2012).

Using the custom-built lettuce protein database, several predicted resistance proteins (RGC1C, RGC2, RGC2C, RGC2K and NBS-LRR resistance-like protein 4T) and a putative ethylene receptor ETR1 (Supporting Information, Table S1) were detected in only lettuce containing internalized *S.* Infantis. This suggests *Salmonella* might have induced the expression of resistance proteins (R proteins), and ethylene might be involved in its regulation. It is known that R proteins can recognize specific effectors secreted by pathogens, leading to the hypersensitive response that prevents the pathogens from growing or spreading inside infected plants (Jones and Dangl, 2006). Ethylene along with salicylic acid and jasmonic acid are the three plant hormones involving signaling and regulating R proteins (Jones and Dangl, 2006).

A few studies investigated plant responses to human pathogens using transcriptomics. Plant pathogenicity-related genes *PR1, PR4*, *PR5* and *DAD1* were induced in lettuce leaf 2 days after *S*. Dublin entered plants through a hydroponic growing medium (Klerks *et al*., 2007,2007). The *PR* genes encode pathogenicity-related proteins, which can be induced as part of systemic acquired resistance (Durrant and Dong, 2004). The expression of *PR1* in alfalfa and *Arabidopsis* was up-regulated by the internalization of *S*. Typhimurium (Iniguez *et al*., 2005; Schikora *et al*., 2001), likely resulting from sensing the T3SS-1 effectors of *Salmonella* (Iniguez *et al*., 2005)*.* In this study, several R proteins were also induced by *Salmonella*, and the secreted effector PipB2 (T3SS-2 effectors) was concurrently detected in internalized *S.* Infantis.

## Concluding remarks

In summary, the global modulation of protein expression revealed *S*. Infantis may utilize alternative carbon sources such as ascorbate upon internalization because the preferred substrate/carbon sources were not available inside lettuce leaves. In the meanwhile, *S*. Infantis produced multiple stress response proteins to cope with the stresses encountered inside plants. On the other hand, proteins involved in lettuce's defense response to bacterium were up-regulated, such as pyruvate dehydrogenase, 2-cys peroxiredoxin and ferredoxin–NADP reductase. Interestingly, the secreted effector PipB2 of *S*. Infantis and R proteins of lettuce were concurrently induced during the interaction between *Salmonella* and lettuce.

## Supporting information

**Fig S1.** The workflow used to separate bacterial proteins and lettuce proteins. **Table S1.** Proteins that were detected in lettuce with internalized *Salmonella* but absent in control lettuce plants.
