# Supplementary material for: Quantitative proteomic analysis of the Salmonella-lettuce interaction
Source: Microb Biotechnol. 2014 Feb 11;7(6):630–7. doi: 10.1111/1751-7915.12114 (PMC4265081; doi:10.1111/1751-7915.12114)
Supplement: Supplementary file 1 [file mbt20007-0630-sd1.docx]

Submitted to *Microbial Biotechnology* special issue on Pathogen in Vegetables

**Quantitative proteomic analysis of the *Salmonella*-lettuce interaction**

Yuping Zhang^1^, Renu Nandakumar^2^, Shannon L. Bartelt-Hunt^1^, Daniel D. Snow^3^, Laurie Hodges^4^, and Xu Li^1,*^

^1^Department of Civil Engineering, ^2^Proteomics and Metabolomics Core Facility, Redox Biology Center, Department of Biochemistry, ^3^School of Natural Resources, ^4^Deptartment of Agronomy & Horticulture, University of Nebraska-Lincoln, Lincoln, NE.

^*^Corresponding author:

844 N. 16th Street, N117 SLNK

Lincoln, NE 68588-6105

Phone: (402) 472-6042

Fax: (402) 472-8934

E-mail: [xuli@unl.edu](mailto:xuli@unl.edu)

Supporting Information: 7 pages including 1 table and 1 figure.

**MATERIALS AND METHODS**

**Lettuce Plant, Salmonella Strain and Infiltration**

Seeds of leafy lettuce (*Lactuca sativa*) cultivar Green Salad Bowl were planted in pasteurized soil (35% Canadian peat, 9% Sharpsburg clay soil, 24% sand, and 32 % small vermiculite) and then grown for 5 weeks in a Biosafety Level 2 greenhouse with 16 h of light at 15-18°C. A *Salmonella* *enterica* serovar Infantis (*S.* Infantis) strain was obtained from Dr. Lisa Durso at the USDA Agroecosystem Management Research Unit. This *S.* Infantis strain was isolated from soil that was amended with cattle manure. *S.* Infantis was cultured in Luria broth (LB) at 37°C for 17 h till mid-stationary phase, and was centrifuge at 6000×g for 10 min at 4°C before the cell pellet was re-suspended in sterile Nanopure water. 300 µL of the resulting bacterial suspension (10^10^ CFU/mL) was infiltrated into the leaves of each treatment plant using a needleless syringe following a published protocol (Katagiri, et al., 2002). Briefly, the tip of a 1-mL needless syringe was pressed against the underside of a leaf. The bacteria suspension was infiltrated into the leaf intracellular space by slowly pressing the plunger while applying gentle counter pressure on the opposite side of the leaf. Same amount of sterile Nanopure water was infiltrated into each control plant. Two biological replicates were included in each treatment. Leaves were harvested 24 h after infiltration. Bacteria on the leaf surface were removed by 5 min of sonication using a Fisher Scientific FS60 ultrasonic cleaner followed by 30 sec of vortexing [modified from Gourion et al (2006)], and the process was repeated for a total of four times. Washed leaves were dried by dabbing with paper towels.

**Protein Extraction**

Washed leaves were ground in a mortar and pestle that were pre-cooled at 4°C. Ground plant tissues were suspended in 5 mL of ice-cold extraction buffer, which was composed of 50 mM ammonium bicarbonate, 8 M urea, and 1.5 mM phenylmethanesulfonylfluoride (PMSF) (Nandakumar, et al., 2011). Plant tissue suspensions were filtered through 11-µm membrane (Waterman Inc., Janesville, WI). Another 5 mL of exaction buffer was used to wash the solids detained on the filter (i.e., plant debris), and the filtrates were combined (Figure S1). The 10 mL filtrate was centrifuged at 10,000×g for 15 min at 4°C and the pellet was used to extract bacterial proteins. The supernatants were combined with the plant debris on filter paper and used for plant protein extraction. Bacterial proteins were extracted by re-suspending the pellet in 1 mL extraction buffer and lysed by a total of 2.5 min of bead beating (0.3 g 0.1 mm diameter glass beads) with 2.5 min interval on ice for every 30 sec bead beating. The cell lysates were centrifuged at 10,000×g for 15 min at 4°C twice to ensure thorough removal of microbial cell debris. The protein concentration in the supernatant was estimated using the BCA protein assay kit (Thermo Scientific, Waltham, MA). The extracted proteins were precipitated overnight with ice cold acetone and centrifuged at 14,000×g for 20 min to recover protein pellets. To extract lettuce protein, plant tissue suspensions were lysed using bead beating (0.3 g 2 mm diameter glass beads). The remaining steps were the same as in bacterial protein extraction.

**Sample Preparation**

Extracted proteins were subjected to in-solution trypsin digestion. Briefly, the proteins were reduced with 10 mM dithiothreitol and alkylated with 40 mM iodoacetamide followed by trypsin (Roche, Indianapolis, IN) (1:50 trypsin: protein ratio) digestion overnight at 37°C. The tryptic peptides were desalted and concentrated using PepClean C-18 spin columns according to manufacturer’s instructions (Thermo scientific, Waltham, MA).

**2D Nano LC-MS/MS Analysis**

2D nano LC-MS/MS was performed with an ultimate 3000 Dionex MDLC system (Dionex Corporation, Sunnyvale, CA) integrated with a nanospray source and LCQ Fleet Ion Trap mass spectrometer (Thermofinnigan, San Jose, CA). The first dimensional separation was performed on a SCX column (Polysulfoethyl, 1mm I.D × 15 cm, 5 μm, 300A, Dionex). 20 μL of samples were loaded onto first dimension SCX column and eluted using a salt gradient (0-600 mM). Selected fractions based on the UV absorbance of the eluted peptides were subjected to second dimension analysis. The second dimension separation incorporated an on-line sample pre-concentration and desalting using a monolithic C_18_ trap column (Pep Map, 300 µm I.D × 5 mm, 100Å, 5 µm, Dionex). The sample was loaded on to the monolithic trap column at a flow rate of 40 µL/min. The desalted peptides was then eluted and separated on a C_18_ Pep Map column (75 µm I.D. × 15 cm, 3 µm, 100Å, New Objective, USA) by applying an acetonitrile (ACN) gradient (ACN plus 0.1% formic acid, 90 minute gradient at a flow rate of 250 nL/min) and were introduced into the mass spectrometer using the nano spray source. The LCQ Fleet mass spectrometer was operated with the following parameters: nano spray voltage, 2.0 kV; heated capillary temperature, 200°C; full scan m/z range, 400-2,000. Data acquisition was done in data dependent mode with 4 MS/MS spectra for every full scan, 5 microscans averaged for full scans and MS/MS scans, a 3 m/z isolation width for MS/MS isolations, and 35% collision energy for collision-induced dissociation.

**Proteomic Data Analysis**

The acquired MS/MS spectra from the bacterial protein samples were searched against the *S*. Typhimurium 14028S database (5323 sequences), and those from the lettuce protein samples against both the *Lactuca sativa* expressed sequence tag (EST) database (128172 sequences) and a custom-made database including *Lactuca sativa* protein sequences (1506 entries) available on NCBI (Cho, et al., 2009). Lettuce proteins identified from the custom-made database was for qualification only. Database search was performed using MASCOT (Version 2.2 Matrix Science, London, UK). The search criteria were set as follows: enzyme: trypsin, missed cleavages: 2; mass: monoisotropic; fixed modification: carbamidomethyl (C); variable modification oxidation (M); peptide tolerance: 1.5Da; MS/MS fragment ion tolerance: 1Da. Probability assessment of peptide assignments and protein identifications were accomplished by Scaffold (Scaffold 4.0 Proteome Software Inc., Portland, OR). The criteria for protein identification included the detection of at least one unique peptide per protein and a protein probability score of ≥90%. Proteins that contained similar peptides and could not be differentiated based on MS/MS analysis alone were grouped to satisfy the principles of parsimony. Relative quantitation of proteins was done by using the label-free method of spectral counting (Liu, et al., 2004) with the normalized spectral counts for each protein. Proteins expressing ≥ 2-fold change in abundance and *p*-value ≤0.05 (Fisher’s exact test) were considered as differentially expressed.

Table S1: Proteins that were detected in lettuce with internalized *Salmonella* but absent in control lettuce plants.

| **Protein name** | **Uniprot Accession** |
| --- | --- |
| 37 kDa chloroplast inner envelope membrane protein | Q9SEC0 |
| ent-kaurene oxidase 1 | B5MEX5 |
| germacrene A synthase LTC2 | Q8S3A5 |
| isopentenyl pyrophosphate:dimethyllallyl pyrophosphate isomerase | Q9M6K6 |
| Lactuca sativa carotenoid cleavage dioxygenase 2 | Q2PHF7 |
| Lactuca sativa zeaxantin epoxidase 1 | Q2PHG3 |
| minichromosome maintenance factor | A7U953 |
| NBS-LRR resistance-like protein 4T | B9UNN6 |
| NBS-LRR resistance-like protein RGC1C, partial | B8XDH9 |
| phenylalanine ammonia-lyase | Q8W2E4 |
| putative deoxyhypusine synthase | A8QVC2 |
| putative ethylene receptor ETR1 | Q5K6M7 |
| resistance protein candidate | O48894 |
| resistance protein candidate | O48893 |
| resistance protein candidate RGC2C | Q9ZSD0 |
| resistance protein candidate RGC2K | Q9ZT68 |
| ribosomal protein S10 | Q9FUT9 |
| RNA polymerase beta subunit | Q56P13 |
| resistance protein RGC2 | Q6Y140 |
| 26S proteasome subunit 7-like protein | A1KXE0 |
| putative ACC synthase 2 | Q84QH5 |
| Gibberellin 3beta-hydroxylase | Q9ZWP9 |


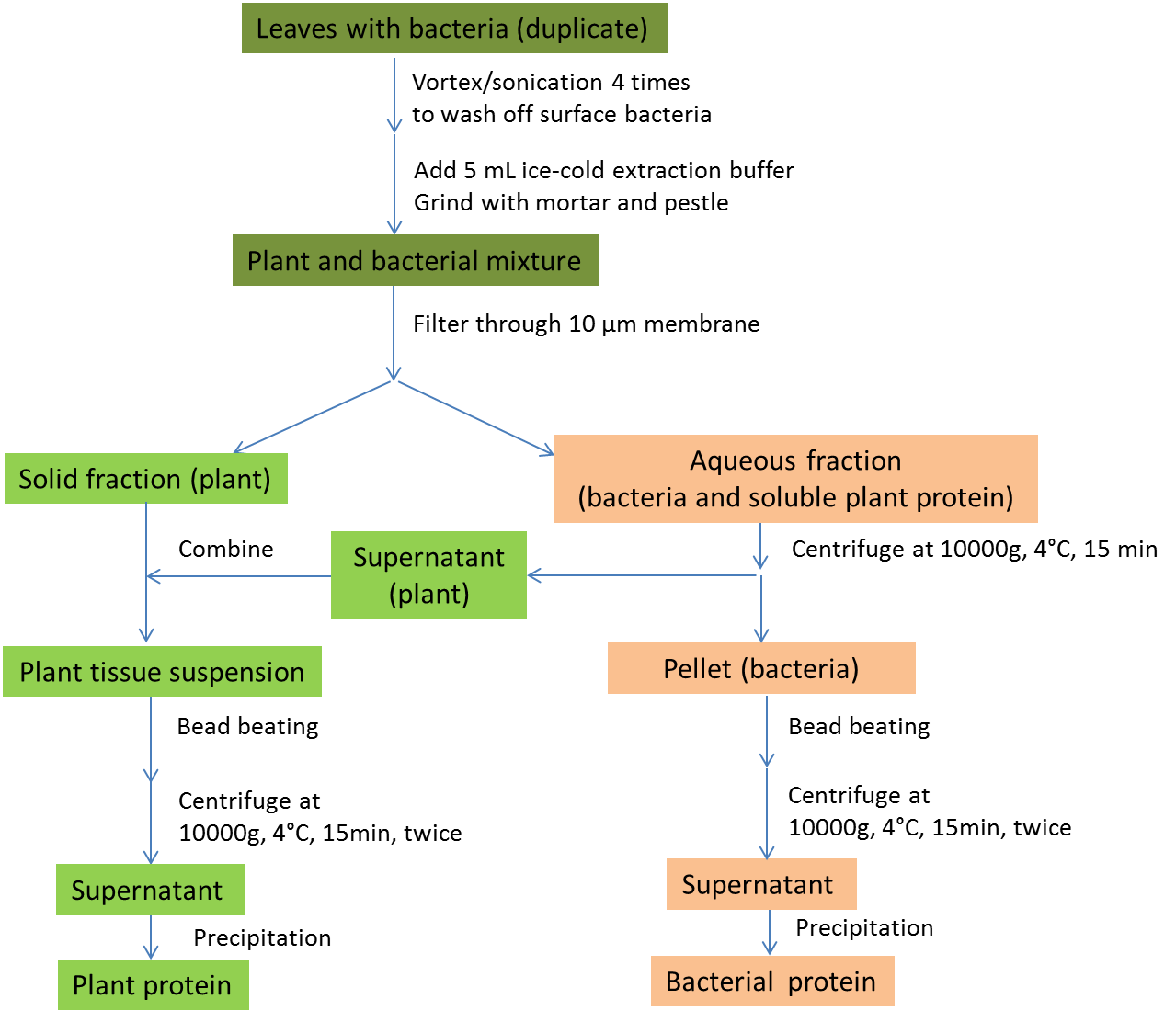


Figure S1. The workflow used to separate bacterial proteins and lettuce proteins.

**REFERENCES**

Cho, W. K., Chen, X.-Y., Uddin, N. M., Rim, Y., Moon, J., Jung, J.-H., et al. (2009) Comprehensive proteome analysis of lettuce latex using multidimensional protein-identification technology, *Phytochemistry* **70**: 570-578.

Gourion, B., Rossignol, M., and Vorholt, J. A. (2006) A proteomic study of Methylobacterium extorquens reveals a response regulator essential for epiphytic growth, *Proceedings of the National Academy of Sciences* **103**: 13186-13191.

Katagiri, F., Thilmony, R., and He, S. Y. (2002) The Arabidopsis thaliana-Pseudomonas syringae interaction, *The Arabidopsis Book/American Society of Plant Biologists* **1**.

Liu, H., Sadygov, R., and Yates, J. (2004) A model for random sampling and estimation of relative protein abundance in shotgun proteomics, *Analytical Chemistry* **76**: 4193-4201.

Nandakumar, R., Santo, C. E., Madayiputhiya, N., and Grass, G. (2011) Quantitative proteomic profiling of the Escherichia coli response to metallic copper surfaces, *Biometals* **24**: 429-444.
